# Recurrence of Hallux Valgus After Modified Lapidus Procedure With Successful Fusion of the Intermetatarsal and Intercuneiform Joints

**DOI:** 10.7759/cureus.15418

**Published:** 2021-06-03

**Authors:** Joseph Long, Jason A Lauf, Brent Whitehead, Nick Cheney, Timothy D Law

**Affiliations:** 1 Medicine, The Ohio State University College of Medicine, Columbus, USA; 2 Orthopedic Surgery, Ohio University Heritage College of Osteopathic Medicine, Dublin, USA; 3 Orthopedic Surgery, Ohio University Heritage College of Osteopathic Medicine, Columbus, USA; 4 Family Medicine, Ohio University Heritage College of Osteopathic Medicine, Athens, USA

**Keywords:** hallux valgus, arthrodesis, modified lapidus, lapidus recurrence, bunion

## Abstract

Background

The Lapidus procedure has become a popular procedure in correcting hallux valgus deformities and has undergone several modifications in an effort to improve the efficacy of the procedure. The senior author modifies this procedure with the addition of an intermetatarsal and intercuneiform fusion. Our hypothesis is that this will improve the procedure outcomes and decrease deformity recurrence.

Methods

We reviewed patient charts who underwent the procedure between 2014 and 2017 performed by the senior author. This yielded 47 reviewable cases, with 34 meeting study criteria. The cases were analyzed for standard hallux valgus measurements (intermetatarsal angle [IMA], hallux valgus angle [HVA]) and fusion on X-ray.

Results

The results of the study showed partial intermetatarsal and intercuneiform fusion failure in seven (20%) cases, and one case where the great toe fell into varus. These cases were excluded. In the remaining cases, there was a statistically significant improvement in the HVA and IMA between the preoperative X-ray and first postoperative X-ray. Additionally, there was no significant difference between HVA and IMA between first and final postoperative radiographs. There was a significant increase in IMA for the fusion failure cases (p=0.001).

Conclusion

Clinically, our findings demonstrate that successful union is possible with low recurrence and complication rates when using this modification of the Lapidus procedure in patients with hallux valgus deformity.

## Introduction

Hallux valgus is a common cause of morbidity in foot and ankle practices, causing pain and decreased quality of life related to the deformity at the first ray [1]. Management of the condition considers factors such as the degree of the deformity and patient symptoms. For patients who have failed conservative management and with moderate-to-severe deformity, the Lapidus and its modified versions have become increasingly popular options to treat the condition operatively [2-6].

Since the first descriptions of the procedure in 1934, several modifications have attempted to improve the efficacy of the procedure, improve the construct stability, and maintain the length of the great toe [6-9]. One of the biggest reasons for these innovations in construct stability is the potential for recurrence of the bunion deformity. Various authors have studied large patient subsets and report recurrences as high as 8% for Lapidus procedures [2,10-14]. To address the concerns for bunion recurrence, the senior author performs a modification to the Lapidus with a fusion of the first and second metatarsal bases and the medial and intermediate cuneiforms, along with the standard Lapidus fusion of the first tarsometatarsal (TMT) joint.

Recent literature has demonstrated the need to consider the more proximal intercuneiforms in the correction of bunion deformity using the Lapidus procedure. Fleming et al, [15] demonstrated that 74% of the patients in their retrospective review had intercuneiform instability with an intraoperative hook test. Galli et al. [16] demonstrated in a cadaveric study that their crossed screw fixation, from the first metatarsal to the intermediate cuneiform, reduced sagittal mobility by an average of 1.3 mm compared to an isolated first TMT fixation. Building on that research, Langan et al. [17] demonstrated with the same construct from Galli et al. [16] that the bunion correction was maintained at a mean follow-up time of nine months.

By fusing the first and second metatarsal bases and the medial and intermediate cuneiforms, the modified Lapidus performed by the senior author (N.C.) takes the aforementioned concepts one step further by attempting to prevent bunion recurrence more proximal to the deformity using a more permanent fixation. The primary goal of hallux valgus surgery is to anatomically correct the deformity and to prevent future recurrence. Our hypothesis is that if the joints fuse properly, the construct will demonstrate no changes in the intermetatarsal angle (IMA) and hallux valgus angle (HVA) after the patient begins to bear weight.

## Materials and methods

Institutional Review Board (IRB) approval was obtained from the senior author’s affiliated institution for chart review of eligible patients. Electronic medical records were reviewed for patients undergoing a modified Lapidus in the senior author’s practice between April 2014 and December 2017. This yielded 47 patients for retrospective chart review. Inclusion criteria for the study analysis were primary bunion correction with preoperative radiographs, non-weightbearing X-ray at the first follow-up, and a follow-up weightbearing X-ray at a minimum of 90 days postoperative. The chart radiographs were then used to determine HVA and IMA by two investigators, and the results were averaged for final data analysis. Fusion of the Lapidus construct was determined by the senior author’s clinical expertise based on clinical and radiographic evaluation.

**Figure 1:**
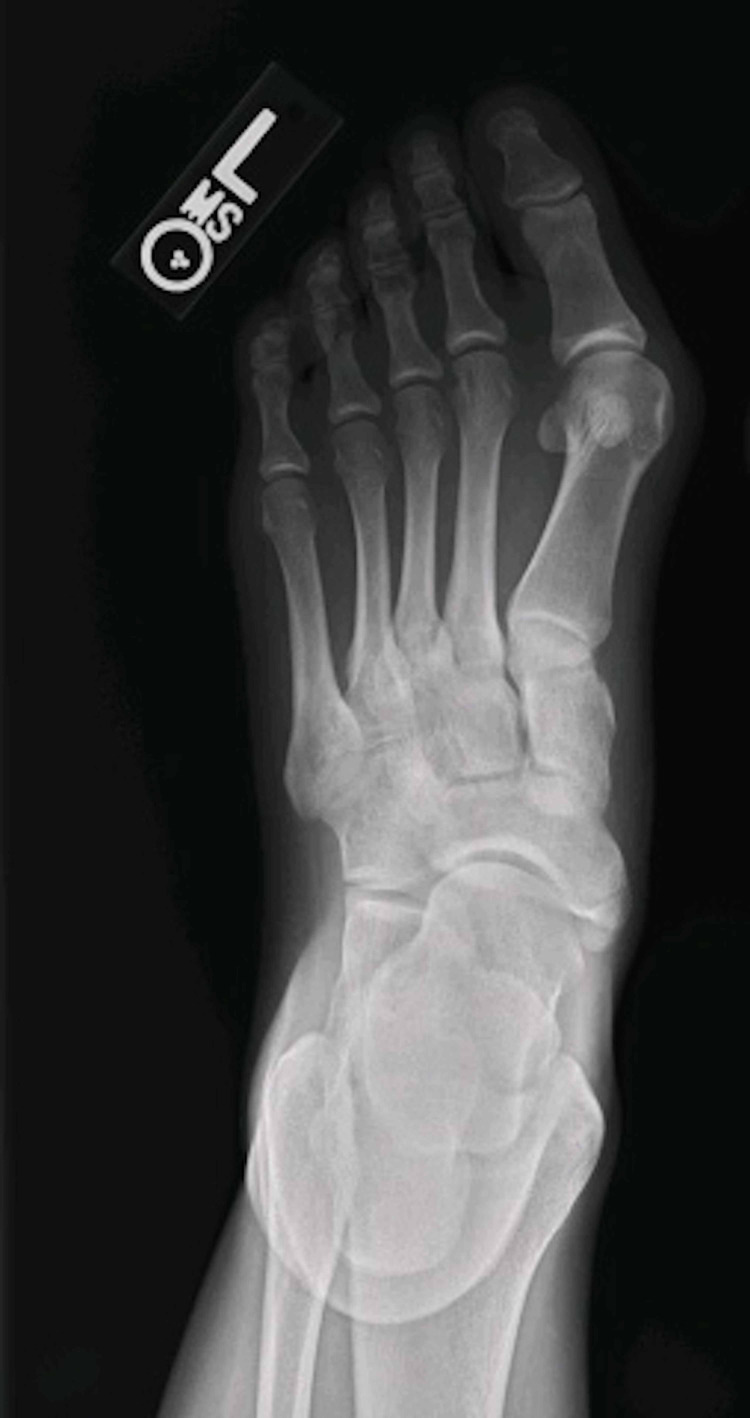
Anteroposterior radiograph demonstrating a preoperative foot with hallux valgus deformity.

**Figure 2:**
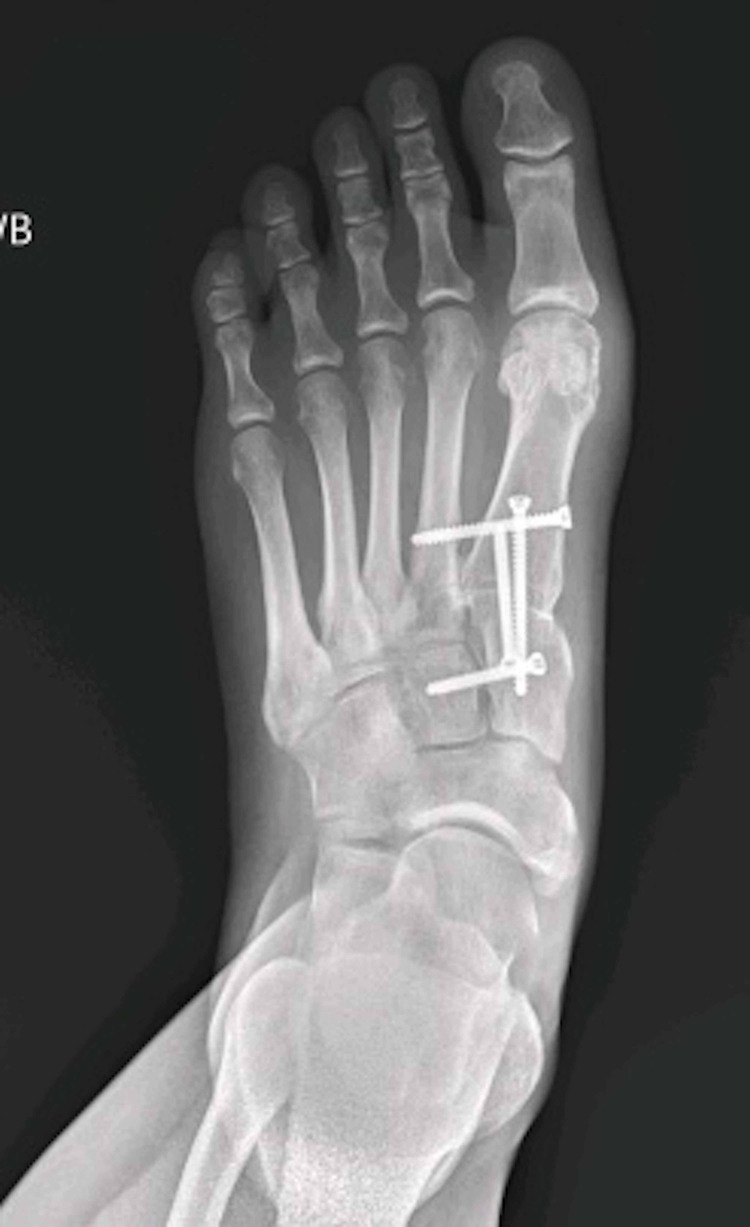
Anteroposterior radiograph demonstrating a postoperative foot from the modified Lapidus procedure.

Indications for the procedure were patients failing conservative methods of treatment, such as shoe orthotics, non-steroidal anti-inflammatories (NSAIDs), and/or a walking boot, and the deformity causing patient distress. A preoperative X-ray demonstrating the hallux valgus deformity can be seen in Figure 1. The surgical procedure for all patients was the same and performed solely by the senior author. A dorsal incision was made over the extensor hallucis longus tendon between the first and second metatarsals and between the medial and intermediate cuneiforms. The first TMT joint was opened, and the cartilage was removed from the joint. Great care is taken to prevent shortening the first ray during the joint preparation as this has demonstrated altered forefoot mechanics and lesser metatarsal overload, and therefore no osteotomies are performed [18]. Soft tissue was then debrided from the intermetatarsal (IMT) and intercuneiform joints and then fenestrated the subchondral bone in the first TMT joint and the medial aspect of the second metatarsal. A distal soft tissue release of the sesamoids and the adductor tendon was completed with resection of prominent dorsomedial bone off the metatarsal head. At this point, a reduction of the TMT joint, as originally described by Hansen [19], was completed and a standard 3.5-mm cortical lag screw was placed across the joint in a distal to proximal direction to provide compression and stability. A second 3.5-mm cortical lag screw was then placed across the joint from proximal to distal. The IMT and intercuneiform joints were manually compressed to close the IMA, and a static 3.5-mm cortical screw was placed from the first metatarsal base into the second metatarsal base to hold the joint compressed and maintain the corrected IMA. A second medial-to-lateral 3.5-mm cortical screw was then placed across the intercuneiform joint to complete the construct. A high-speed burr was then used to decorticate the bone at the confluence of the metatarsal bases and the cuneiforms, and bone autograft harvested from the posterior lateral calcaneus is placed to assist in healing of the entire complex. All patients received the same bone autograft for the procedure. The HVA and IMA were measured to assure deformity correction. The final outcome of the procedure can be seen in Figure 2.

Postoperatively, patients were placed into a boot and remained non-weightbearing for eight weeks. After that time, they were transitioned to weightbearing status using a one-month weight progression protocol. X-rays were obtained for patients preoperatively, at two weeks postoperatively, at eight weeks postoperatively, at 12 weeks postoperatively, and as needed after 12 weeks. The preoperative, 12 weeks postoperative, and as needed radiographs are obtained with the patient weightbearing. The two weeks postoperative and eight weeks postoperative radiographs are non-weightbearing as per the protocol. If the patient has X-ray proven fusion and no clinical symptoms, the patient is released to follow-up on an as-needed basis.

Statistical analysis was composed of a two-tailed t-test to compare statistical significance between preoperative and first postoperative, first and final postoperative, and preoperative and final postoperative values.

## Results

Of the 47 cases that were eligible for chart review, 34 cases met the study criteria. Of the 13 cases that did not qualify, 11 cases were due to inadequate follow-up data/radiographs, and two were revision cases. Eight cases were excluded from their respective group data analysis: seven (21%) cases with radiographic evidence of IMT and intercuneiform nonunion and one (3%) case that fell into hallux varus. The seven cases of nonunion were analyzed on their own. All of the patients achieved fusion at the TMT joint. The 34 cases that met the study criteria resulted in 26 cases used in statistical analysis. Demographic data included six men aged 43.7±16.1 years and 20 women aged 57.2±11.6 years. The mean radiographic follow-up time was 10.5±8.9 months (range: 3.1-41.5 months). The breakdown of concomitant procedures performed on all patients can be seen in Table 1.

**Table 1:** Concurrent procedures of the cases that met study criteria.

Procedure	Study Group	Fusion Failure
Lapidus only	3	1
Hammertoe	14	2
Gastrocnemius recession	29	6
Cyst curettage	1	0
Calcaneal cuboid joint debridement	1	0
Tendon debridement	1	0
Tendon transfer	2	1
Hallux cheilectomy	1	0
Tendon tenolysis	2	0
Hardware removal	1	0

For these 26 cases, the average IMA measurement was 13.8°±3.1°, 7.1°±2.4°, and 8.4°±3.7°, and HVA measurements were 27.8°±9.2°, 17.5°±6.8°, and 18.4°±8.7° for each consecutive time point, respectively. For the fusion failure group (n=7), the average IMA measurements were 13.0°±2.4°, 8.1°±2.1°, and 12.6°±4.1°, and the HVA measurements were 29.3°±14.3°, 18.6°±9.8°, and 21.7°±12.1° for each consecutive time point, respectively. This information can be seen in Table 2.

**Table 2:** Angle and statistical measurements of the 34 cases that met study criteria. IMA, intermetatarsal angle; HVA, hallux valgus angle

	Study Group (n=26)	Fusion Failures (n=7)
Preoperative IMA	13.8°±3.1°	13.0°±2.4°
First postoperative IMA	7.1°±2.4°	8.1°±2.1°
Final postoperative IMA	8.4°±3.7°	12.6°±4.1°
Preoperative HVA	27.8°±9.2°	29.3°±14.3°
First postoperative HVA	17.5°±6.8°	18.6°±9.8°
Final postoperative HVA	18.4°±8.7°	21.7°±12.1°
IMA preoperative and first postoperative p-value	<0.001	0.0013
IMA first postoperative and final postoperative p-value	0.26	0.007
HVA preoperative and first postoperative p-value	<0.001	0.59
HVA first postoperative and final postoperative p-value	0.77	0.49

In the study group, the difference in IMA and HVA was significant between the preoperative and first postoperative X-ray (p<0.001 and p<0.001, respectively). However, there was not a significant difference in the IMA (p=0.26) or HVA (p=0.77) between the first and final postoperative radiographs. Of note, the cases with IMT fusion failure saw a statistically significant increase in IMA measurements between postoperative radiographs (p=0.001) but no statistically significant changes in HVA postoperative measurements (p=0.49).

## Discussion

Based on the data obtained and analyzed in this study, the modified Lapidus performed by the senior author is a viable option to prevent bunion recurrence. To support this, we saw that the IMA and HVA did not significantly change in the subset of patients with confirmed fusion of the IMT joint based on postoperative images. Additionally, the importance of successful IMT fusion in preventing deformity recurrence was further highlighted in our study by the fact that significant postoperative increase in HVA and IMA occurred in cases with nonunion. A study by Langan et al. [17] evaluated a construct similar to that of the senior author. Langan et al. also included an intercuneiform fusion in order to improve foot stability. There was a significant reduction in the preoperative and postoperative IMA and HVA measurements, without a significant change postoperatively in cases of joint fusion [17]. Their study helps to validate the stability of the construct used by the senior author. In the end, a fusion between the first and second metatarsals and the medial and middle cuneiforms should prevent any rotation, translation, or angulation of the first ray. In theory, this should be able to prevent recurrence indefinitely if the fusion heals successfully and assuming the deformity was reduced correctly. With that thought, the length of time for follow-up might be less important than the fusion healing completely.

One of the concerning findings of our study was the high nonunion rate (7/34; 21%) of the IMT and intercuneiform joints seen in the cohort. This rate is on the borderline of what is acceptable for arthrodesis, but it is difficult to interpret without additional patient demographic and lifestyle data that were not available for analysis. This is significant because we believe that the intercuneiform and IMT joint fusion variation can eliminate the recurrence of the hallux valgus deformity. Our patients achieved 100% fusion rate of the first TMT joint, which is seen in a standard Lapidus procedure. A study performed by Thompson et al. [20] also specifically looked at the rate of TMT joint fusion. They found a 1.5% nonunion rate at this joint, which follows our results of 0% TMT joint nonunion [20]. When the construct heals, it prevents an increase in IMA/HVA or recurrent pronation of the IMT/intercuneiform joints, which would in theory increase the stability of the medial column. There have been several studies performed examining the nonunion rate following a modified Lapidus procedure as a whole [13,20-24]. Nonunion in these studies ranged from 2.2% in 136 patients by King et al. [13] to 12% in 32 feet by McInnes and Bouché [21].

Potential reasons for the high nonunion rate in the current study include patient characteristics, the degree of difficulty of the added IMT fusion, and biomechanical factors outside the study scope. Prissel et al. [22] examined several demographic factors that were compared between union and nonunion patients. They discovered no significant differences in gender, age, body mass index (BMI), nicotine use, or diabetes [22]. These several factors that were not within the study scope could be included in future analyses. In terms of procedure difficulty, Maenohara et al. [25] used CT scans to create recommendations with some clinical data for potential locations of intercuneiform screws for arthrodesis. Their study highlights the complex nature of the anatomy seen in this area and gives credence to the difficulty this screw placement has. However, we believe that the intercuneiform screw by itself is unable to maintain the deformity reduction, unless the joints are already fused. Based on the results of this study, we believe that the intercuneiform screw should not be reviewed without the corresponding IMT joint if there has been successful fusion at the joints.

Possible benefits of this intercuneiform screw have conflicting support in the current literature. The intercuneiform screw has support from the aforementioned studies, which demonstrated the value of the additional construct stability more proximally [15-17]. However, a screw in this area has been studied biomechanically by Feilmeier et al. [26] and does not increase stability of the first ray in the transverse or coronal planes in a TMT arthrodesis. Therefore, additional research should be conducted to evaluate the changes in foot biomechanics and Lapidus construct stability after intercuneiform screw placement. However, our modification includes the IMT joint and not just the intercuneiform joint. Theoretically, if the bases of the first and second metatarsals along with the intercuneiform joints have fused, the ability for any proximal motion to affect recurrence should be neutralized. It goes beyond just TMT hypermobility or intercuneiform joint instability in an effort to prevent recurrence by addressing all proximal motion. Previous studies review solely the TMT joint or intercuneiform joints. Our study combines these two joints as well as the IMT joint to improve proximal stability. The results showed near-zero deformity recurrence rate when the IMT joint heals.

Some of the limitations of our study design include small sample size, medium length of follow-up, and the retrospective nature of the study. With only 34 usable cases to compare, there could be outliers such as with the nonunion cases, which confound the study results. Additional research with more patients could identify these outliers and attempt to identify whether the nonunion rate is truly higher or not. The follow-up time of our retrospective study was limited to patient data collected previously for only clinical purposes, which limits how much information could be gathered and the length of time we could follow patients. Further studies should be performed assessing long-term follow-up of patients performed using this modified technique. Further studies should also be performed examining possible risk factors leading to nonunion. Another limitation would be the use of patient-reported data, in order to gather subjective data from patients about their foot symptoms, as these were what drove them toward surgical correction in the first place.

## Conclusions

In conclusion, our study shows that the modified Lapidus procedure is a viable option for treating hallux valgus. The results show that joint fusion across the intercuneiform and IMT joints demonstrates a recurrence rate lower than results described from previous studies. The study shows no recurrence with radiographic evidence of successful healing of the first TMT joint, and successful IMT and intercuneiform joint fusion. The study also shows that HVA and IMA can increase postoperatively (i.e., increased recurrence rate) when there is no IMT and intercuneiform joint fusion, even though there was successful first TMT joint healing.
